# Target-Based Anticancer Indole Derivatives for the Development of Anti-Glioblastoma Agents

**DOI:** 10.3390/molecules28062587

**Published:** 2023-03-13

**Authors:** Silvia Salerno, Elisabetta Barresi, Emma Baglini, Valeria Poggetti, Federico Da Settimo, Sabrina Taliani

**Affiliations:** Department of Pharmacy, University of Pisa, Via Bonanno 6, 56126 Pisa, Italy

**Keywords:** indole, brain tumor, glioblastoma, heterocyclic chemistry

## Abstract

Glioblastoma (GBM) is the most aggressive and frequent primary brain tumor, with a poor prognosis and the highest mortality rate. Currently, GBM therapy consists of surgical resection of the tumor, radiotherapy, and adjuvant chemotherapy with temozolomide. Consistently, there are poor treatment options and only modest anticancer efficacy is achieved; therefore, there is still a need for the development of new effective therapies for GBM. Indole is considered one of the most privileged scaffolds in heterocyclic chemistry, so it may serve as an effective probe for the development of new drug candidates against challenging diseases, including GBM. This review analyzes the therapeutic benefit and clinical development of novel indole-based derivatives investigated as promising anti-GBM agents. The existing indole-based compounds which are in the pre-clinical and clinical stages of development against GBM are reported, with particular reference to the most recent advances between 2013 and 2022. The main mechanisms of action underlying their anti-GBM efficacy, such as protein kinase, tubulin and p53 pathway inhibition, are also discussed. The final goal is to pave the way for medicinal chemists in the future design and development of novel effective indole-based anti-GBM agents.

## 1. Introduction

In recent decades, brain tumors have an increasingly frequent incidence [1]. The average annual incidence rate of all brain tumors in the years 2013–2017 was 23.79 per 100,000, with a higher rate in females than males (26.31 versus 21.09), being 7.08 per 100,000 the average annual incidence rate of primary malignant brain tumors. It has been estimated that 83,830 new cases of primary brain tumors and other central nervous system (CNS) tumors were diagnosed in the United States in 2020 and this includes an expected 24,970 primary malignant tumors [2]. In 2020, worldwide, about 308,102 people were diagnosed with a primary tumor of the brain or spinal cord. Finally, in the United States, in 2022, about 25,050 cases (14,170 men and 10,880 women) of primary brain and spinal cord tumors were diagnosed [3].

Primary brain tumors are mainly termed gliomas and, based on their cellular origin, they can be classified in astrocytic tumors, oligodendrogliomas, mixed gliomas and ependymomas [4]. Glioblastoma (GBM) is a solid tumor of the brain or spinal cord originating from a microglial cell subtype, termed astrocytes. Brain tumors have also been classified, from the mildest to the most aggressive, in four grades [5]: pilocytic astrocytoma (I), diffuse astrocytoma (II), anaplastic astrocytoma (III), and GBM (IV). GBM represents the most aggressive brain tumor, mainly due to its ability to penetrate healthy tissues thanks to the maintenance of sustained angiogenesis. GBM is also the most frequent primary brain tumor, accounting for approximately 15% of all primary brain tumors in adults and with a worldwide incidence of less than 10 per 100,000 people [6,7,8,9]. Furthermore, among the various brain tumors, GBM also has a poor prognosis and higher mortality rate [1,10].

Currently, the GBM therapeutic protocol consists of surgical resection, radiotherapy, and chemotherapy with temozolomide. Complete surgical resection of the tumor improves survival rates, but it is extremely difficult, due to infiltration into the surrounding tissues and, as a result, GBM recurs, often leading to the death of patients [11,12]. Both radiotherapy and temozolomide induce DNA double strand breaks thus activating apoptosis to achieve GBM cell death. In clinical trials, patients with GBM still have an average survival of approximately 15–18 months and a time to recurrence of approximately 7 months, with a 5-year survival of less than 10% or even shorter in older patients [13]. In addition, once GBM has recurred, the mean overall survival was approximately 24–44 weeks [14]. There are poor treatment options for inexorable recurrence, and with chemotherapy only modest anticancer activity is achieved [15]. The standard therapy for recurrent GBM are DNA alkylating drugs, such as lomustine and carmustine, and bevacizumab, an antiangiogenic agent approved by the U.S. Food and Drug Administration (FDA) for the care of relapsing GBM [16]. Moreover, the over-activation or dysregulation of several signaling pathways in GBM can lead to the uncontrolled growth of primary and recurrent tumors [17]. Therefore, there is still a need for the development of new targeted therapeutic treatments that are effective in GBM [18,19].

The research in this field is based on the evidence that the deregulation of three main pathways have been highlighted in GBM, namely the receptor tyrosine kinases (RTKs)/Ras/PI3K pathway (altered in 88% of GBM patients), the p53 pathway (altered in 87% of GBM patients), and the retinoblastoma (RB) protein pathway (altered in 78% of GBM patients) [17].

Heterocyclic compounds, containing at least one heteroatom such as oxygen, nitrogen or sulfur, can be considered both acceptors and donors of hydrogen bonds, so that they can effectively bind to various molecular targets eliciting numerous pharmacological effects [20,21]. Moreover, they are able to alter the liposolubility and therefore the aqueous solubility of drug molecules, thus achieving suitable and remarkable pharmacotherapeutic properties [22]. In this respect, nitrogen-containing heterocycles, as recently highlighted by the FDA, are considered the most relevant heterocyclic nuclei in drug design [23] and, in particular, indole has gained considerable interest in the last decades due to its multiple bioactivities [24].

Indole is a naturally occurring heterocycle, in which the five-membered electron-rich pyrrole ring and the benzene portion confer electronic and steric properties so as to make it a hetero-annulate nucleus widely exploited in drug discovery campaigns. Accordingly, thanks also to its bioavailability and pharmacological activities, indole is considered one of the most privileged scaffolds in medicinal chemistry with potential for different therapeutic applications [25].

In the field of anticancer drugs, the approval and use of alkaloids containing indole, such as vincristine and vinblastine, inspired the researchers to design and synthesize many other indole-based compounds, in order to obtain structurally different leads with distinctive mechanisms of antitumor action [20,26,27].

The following section focuses on the recent advances (2013–2022) made in the design of anti-GMB indole-based compounds, that may act through the modulation of various molecular targets such as kinases, tubulin and p53.

## 2. Indole Derivatives as Anti-Glioblastoma Agents

Despite several studies highlighted many pharmacological applications of indole-based compounds as anticancer agents [28,29,30,31,32], there are only few reviews describing the recent advances in the discovery of small molecules for GBM treatment [14,33] and none of these are specifically focused on the development of indole-based compounds for the treatment of GBM. This report constitutes a brief review focused on the indole-based derivatives in subclinical and clinical stages of development as anti-GBM agents, with particular reference to the most recent medicinal chemistry advances reported between 2013 and 2022. When possible, structure–activity relationships (SARs) are also discussed.

### 2.1. Indole-Based Anti-GBM Compounds Acting on Receptor Tyrosine Kinases (RTKs)/Ras/PI3K Pathway

In GBM, the commonly mutated or amplified RTKs are the epidermal growth factor receptor (EGFR) (57.4%), platelet-derived growth factor receptor (PDGFR) (13.1%), mesenchymal epithelial transition (MET) (1.6%), and fibroblast growth factor receptor 2/3 (FGFR2/3) (3.2%). Actually, these altered RTKs affect most of the cellular pathways and processes, including apoptosis, cell growth and survival [34].

#### 2.1.1. Indole-Based PDGFR and VEGFR Inhibitors 

**Chart 1:**
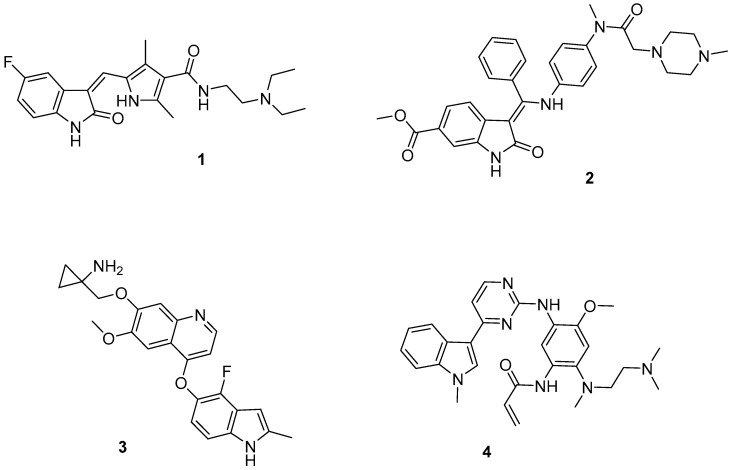
Indole-based PDGFR/VEGFR inhibitors (**1**–**3**) and EGFR inhibitor (**4**).

It has been reported that GBM stem cell (GSC) growth is supported by abnormal angiogenesis and that these cancer cells can also transdifferentiate directly into endothelial cells or growth factors, such as vascular endothelial growth factor (VEGF), thus regulating the tumor vascular system [35]. Moreover, the platelet-derived growth factor (PDGF) and its receptor (PDGFR) are also overexpressed in approximately 16% of GBM. Therefore, a simultaneous administration of VEGF and PDGF pathway inhibitors could produce a beneficial effect in chemoradiotherapy protocols [36]. Actually, several VEGFR/PDGFR inhibitors are marketed for cancer therapy and have been exploited for the treatment of GBM and, among them, the indole derivative Sunitinib (Sutent, Pfizer) **1** (Chart 1), already approved in 2006 by the FDA for the treatment of renal cell carcinoma and gastrointestinal stromal tumor [14]. In preclinical studies, Sunitinib **1**, despite its limited brain penetration in mice, showed a direct antiproliferative effect and prolonged survival in GBM mice [37]. Unfortunately, clinical evaluations showed that **1** did not appear to be particularly useful in patients with GBM [14]. In fact, in a phase II trial, single-agent Sunitinib **1** with continuous high dosing (37.5 mg) did not prolong the six-month progression-free survival in patients with recurrent GBM. This is mainly due to the toxicity of Sunitinib **1** which prevented continued treatment beyond 3 weeks (necessary to achieve optimal pharmacokinetics), thus resulting in suboptimal drug exposure for these GBM patients [37].

Analogously, Nintedanib (Cyendiv, Boehringer-Ingelheim) **2** (Chart 1), a potent triple VEGFR, PDGFR, and FGFR angiokinase inhibitor, does not elicit beneficial effects in GBM, likely due to its limited CNS penetration. Anlotinib **3** (Chart 1) is an oral, selective VEGFR2 inhibitor with significantly fewer side effects than **1** [38], approved in China for the therapeutic treatment of patients with non-small cell lung cancer (NSCLC) [39]. A clinical case of recurrent GBM revealed a reduction in tumor size 26 days after treatment with compound **3**, although the tumor continued to progress 2 months after the therapy with **3** [40]. To date, a 50-participant clinical trial evaluating the efficacy of compound **3** in the treatment of recurrent GBM is being recruited (NCT04004975).

#### 2.1.2. Indole-Based EGFR Inhibitors

Osimertinib (Tagrisso, Astra Zeneca) **4** (Chart 1), an oral EGFR inhibitor with very high brain penetration properties, was able, in preclinical evaluation, to significantly inhibit the growth of six GBM cell lines [41]. In an in vivo GBM model, compound **4** was shown to significantly inhibit tumor growth and to prolong the survival time of animals. A patient with multifocal GBM characterized by multiple EGFR mutations had a complete response to compound **4** at only one of the tumor sites [42]. This case highlights the heterogeneity of this tumor, which is still a major challenge for EGFR-targeted GBM therapy, together with the limited brain permeability of most TKIs and the low specificity of small molecules inhibiting GBM-related EGFR mutations [14].

#### 2.1.3. Indole-Based PI3K/AKT/mTOR Pathway Inhibitors 

Among RTK pathways, PI3K/AKT/mTOR is one of the pivotal triggers of the development of glioma, with a lack of apoptosis and consequent unregulated cell growth [43,44]. Moreover, in clinical analysis of patients with GBM, the PI3K pathway is abnormally activated, resulting in a poor prognosis [45].

**Chart 2:**
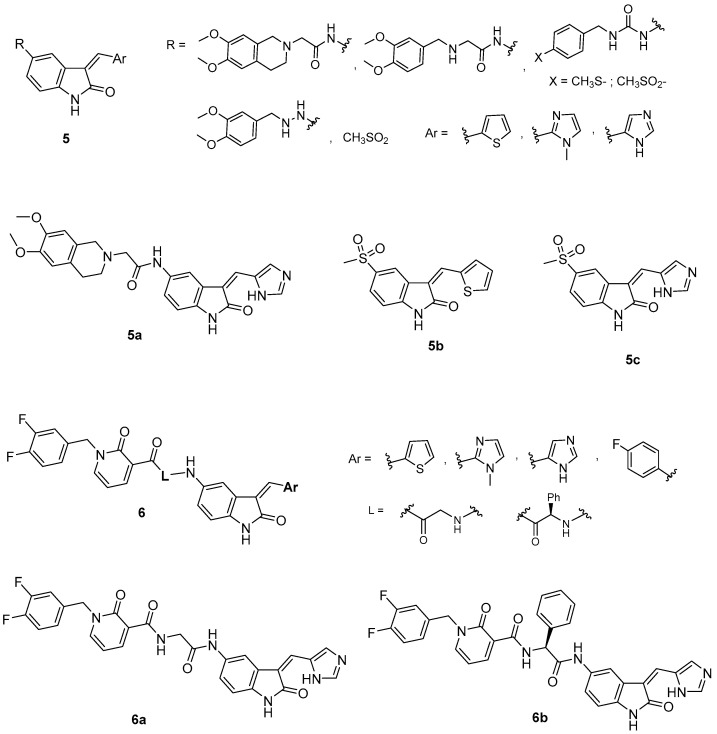
Indole-based PI3K/AKT/mTOR pathway inhibitors (**5**–**6**).

**Figure 1:**
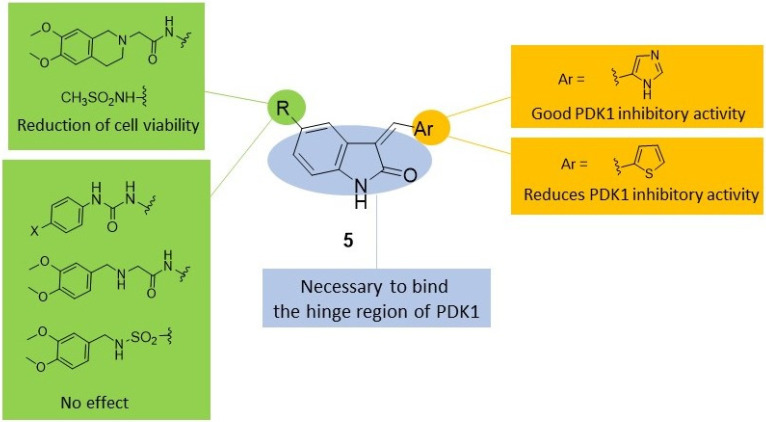
SAR [49] summary for PDK1 inhibitory activity and effects induced on ASPC1 cell viability by indole-based derivatives **5**.

In the search for new PDK1/Akt inhibitors as a promising treatment for GBM, the oxindole nucleus present in different PI3K/Akt pathway inhibitors [46,47,48], a small family of 2-oxindole derivatives of general formula **5** (Chart 2, Figure 1) were synthesized and evaluated in vitro [49]. In particular, the substitution pattern at the 5-position was investigated with regard to the activity on PDK1 and Akt kinases; several side chains were introduced at this position, including the 3,4-dimethoxytetrahydroisoquinoline-N-acetamide, 3,4-dimethoxybenzylamino-N-acetamido group (a group with greater flexibility and conformational freedom), substituents with an electron-rich linker like the arylurea- and arylmethyleneamino-sulfonyl moieties, or with a less hindered group, like the methanesulphonylamido one. Human pancreatic adenocarcinoma cell line ASPC1 [50] was used to preliminarily evaluate the cytotoxicity of the sixteen synthesized compounds, as this cell line is a suitable model to study the inhibition of the PI3K-AKT pathway.

The results obtained showed that, within compounds bearing the 3,4-dimethoxytetrahydroisoquinoline alkylamido side chain, only derivative **5a** (Chart 2) diminished the cell viability to about 68%. The substitution of this chain with thiomethylphenylureido or methylsulphonylphenylureido groups resulted in deleterious activity, whereas replacement with a small electron rich group, such as the methanesulphonamido group (compounds **5b–c**, Chart 2), maintained a comparable effect on cell viability with respect to compound **5a** (68% for **5b** and 74% for **5c**). The substitution of the 3,4-dimethoxytetrahydroisoquinoline-N-acetamido group with a more flexible and a more conformationally free group, such as the 3,4-dimethoxybenzylamino-N-acetamido and 3,4-dimethoxyaminosulphonyl moieties, respectively, diminished the antiproliferative potency (>85%). Compounds **5a–c** were further investigated on the PDK1/Akt pathway, and only **5a** (Chart 2) was shown to inhibit PDK1 kinase and also other downstream effectors essential for GBM-derived stem cell survival.

Moreover, compound **5a** was able to effectively reduce, in a concentration-dependent manner, the viability of two different human GBM cell lines, ANGMS-CSS and U118MG (GI_50_ values ranging from 3.98 to 29.93 μM), which are resistant to conventional chemotherapeutic agents, such as temozolomide. Of note, based on its drug likeness and CNS multiparameter optimization (CNS MPO) score, compound **5a** should have very good blood–brain barrier (BBB) penetration properties [49].

**Figure 2:**
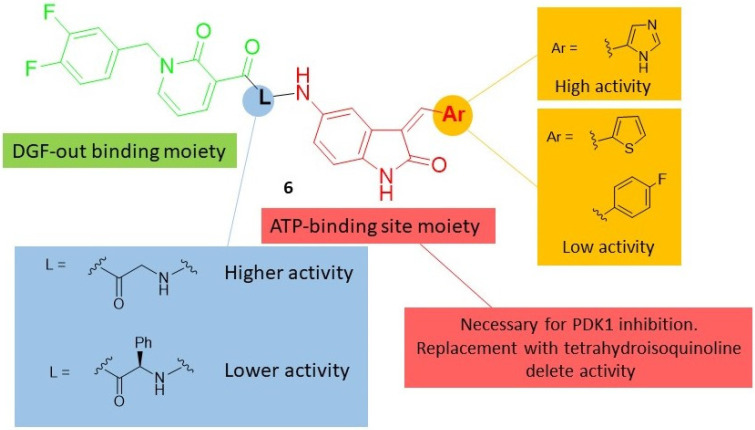
SAR [51] summary for PDK1 inhibition activity of indole-based derivatives **6**.

Later, some new 2-oxo-indole-pyridonyl derivatives, of general formula **6** (Chart 2, Figure 2), with improved activity were reported. The preliminary PDK1 screening of these compounds evidenced that the combination of the 2-oxindole framework with the 2-pyridonyl one is important for the kinase affinity. Indeed, replacement of the 2-oxoindole core with the tetrahydroisoquinoline moiety furnished compounds that were inactive against PDK1. Within the pyridonyl series, the substitution at the 3-position of the 2-oxoindole moiety significantly affects the PDK1 affinity. Notably, the (1H-imidazol-5-yl)methylene group improves the potency against PDK1 in the series of directly fused derivatives, as well as in those with a spacer (L) between the two heterocycles. Even the spacer (glycine or phenylglycine) significantly affects the activity, indicating the glycine is the best linker. In particular, the oxoindolepyridonyl derivative **6a** (Chart 2) was found to be the best PDK1 inhibitor with an IC_50_ value of 0.112 μM in the PDK1 inhibition assay. Moreover, compound **6a** was able to inhibit U87MG cells and GSCs proliferation with an IC_50_ value of 449.7 nM and 3.36 nM, respectively [51].

The simultaneous inhibition of PDK1 and aurora kinase A (AurA), both playing a crucial role in cell survival, may be an innovative strategy to treat GBM, overcoming its resistance and recurrence. In this context, the 1-phenyl derivative of **6a**, namely **6b**, (Chart 2), thanks to its dual PDK1/Aurora Kinase A inhibitory activity was able to induce apoptosis in U87MG cells with a decrease in cell proliferation and migration. Moreover, **6b** inhibited the formation and proliferation of GSCs isolated from U87MG cells with an IC_50_ value of 8.3 nM. A similar behavior was also observed against GSCs isolated from U343MG and ANGM-CSS cells, as compound **6b** reduced their proliferation with IC_50_ values of 0.05 and 0.04 μM, respectively [52].

### 2.2. Indole-Based Tubulin Polymerization Inhibitors

Recent studies showed that GBM cells are particularly sensitive to mitotic destruction compared to healthy cells, so the use of therapies that disrupt microtubule (MT) assembly could represent a promising strategy to treat this type of cancer [53]. In support of this, devices that generate electromagnetic fields and work by disrupting MTs have shown significant efficacy and have been approved as new treatments for GBM [54]. In this context, antitubulin agents could have anticancer efficacy in patients with GBM. However, unfortunately, most antitubulin agents do not easily cross the BBB [55].

**Chart 3:**
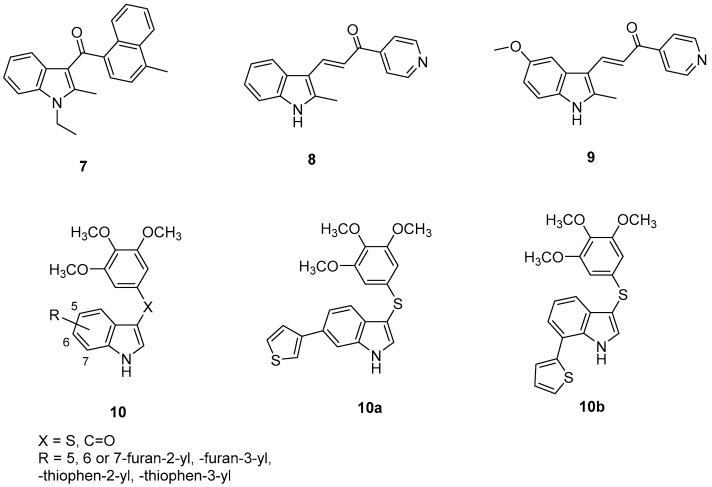
Indole-based tubulin polymerization inhibitors (**7–10**) in GBM.

A library of 2-aroylindole agents that interact with the colchicine-binding site, inhibiting tubulin polymerization, and are insensitive to multidrug resistance efflux pumps that can hinder brain penetration, was developed [56,57,58]. Afterwards, a series of alkylindole compounds able to kill glioma cells in vitro [59] was designed, and, among this class, ST-11 (**7**, Chart 3), acts directly on MT, disturbing MT dynamics and contributing to aberrant spindle formation. ST-11, **7** showed efficacy in killing GBM cells U251, A172, and U87 and the GSC lines BT74, MGG4, and MGG8 at micromolar concentrations (EC_50_ from 2.4 to 8.6 μM) [60]. Moreover, in vivo studies showed that, unlike current antitubulin agents, compound **7** was able to readily cross the BBB and, in a mouse model of GBM, it was able to reduce tumor volume without evident toxicity [60].

Another alkylindole derivative, compound **8** (Chart 3), was a promising anti-GBM agent [61] with a mechanism of induction of methuosis (a nonapoptotic cell death associated with vacuolization in both temozolomide-resistant and nonresistant GBM cells) [62]. Compound **8,** at 1 μM concentration, was able to induce transient effects in U251 GBM cell morphology within 4 h, but most of the vacuoles were dispelled in 24 h, while they remained persistent for 24 h and beyond at higher concentration (10 μM). Moreover, despite its therapeutic potential, compound **8** presented a limited solubility in aqueous solutions [61]. Previous SAR studies on this class of compounds evidenced the availability of some flexibility at the 5-position of the indole ring, so that modifications were made at this position with groups that would increase polarity at physiological pH, while maintaining a methyl at the 2-position. This study led to the development of a more potent derivative, compound **9** (Chart 3) [63], able to inhibit U251 GBM cells’ proliferation with a lower IC_50_ value (1.9 μM) than compound **8** (4.8 μM). Further studies on alkylindole derivatives indicated that some particular substitutions at the indole 2-position could cause microtubule disruption, instead of methuosis, resulting in less potent compounds [63].

**Figure 3:**
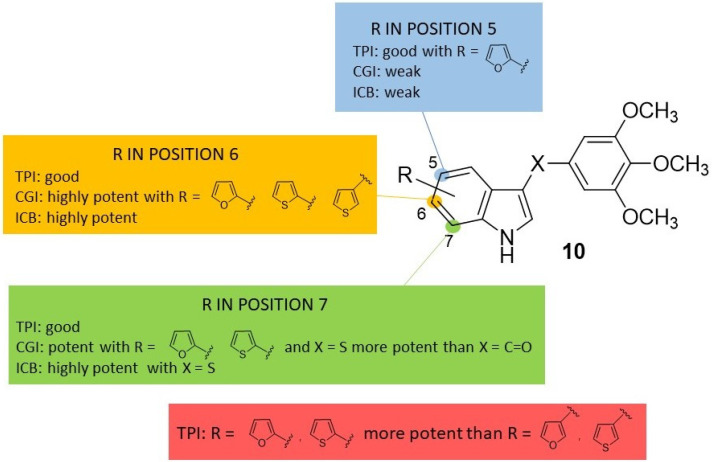
SAR [64] summary for tubulin polymerization inhibition (TPI), MCF-7 cell growth inhibition (CGI) and inhibition of colchicine binding (ICB) of indole based derivatives **10**.

In 2018, twenty new 3-arylthio- and 3-aroyl-1H-indole derivatives (general formula **10**, Chart 3, Figure 3) substituted at the 5-, 6- or 7-position of the indole scaffold with a heterocyclic ring were designed [64]. Among them, some 6- and 7-heterocyclyl-1H-indole compounds were able to host tubulin polymerization, by inhibiting the binding of colchicine to tubulin at submicromolar concentrations, and the growth of MCF-7 cancer cells with IC_50_ values in the range of 150–350 nM. Two representative very potent derivatives of the 6- and 7-heterocyclyl-1H-indoles, **10a** (R = -thiophen-3-yl) and **10b** (R = -thiophen-2-yl), respectively, resulted in the most interesting compounds of all the series. Compound **10a** efficaciously inhibited tubulin polymerization at 50 nM, its activity comparable to that of vinblastine (a breast cancer drug, inhibitor of tubulin polymerization), while compound **10b** resulted in affecting the mitotic spindle structural organization, without fully inhibiting tubulin polymerization, at a concentration of 100 nM. Compounds **10a** and **10b** potently inhibited proliferation of a panel of cancer cells and the NCI/ADR-RES multidrug resistant cell line at low nanomolar concentrations. Moreover, in cell cycle analysis, compound **10a** became effective at 20 nM and induced 77% G2/M arrest at 50 nM, and resulted in being a potent inhibitor of the U87MG cell line at 16 nM concentration, being more active than the yet reported arylthioindoles featuring the imidazole-1-yl or pyridine-4-yl moiety at 2-position of the indole [65].

**Chart 4:**
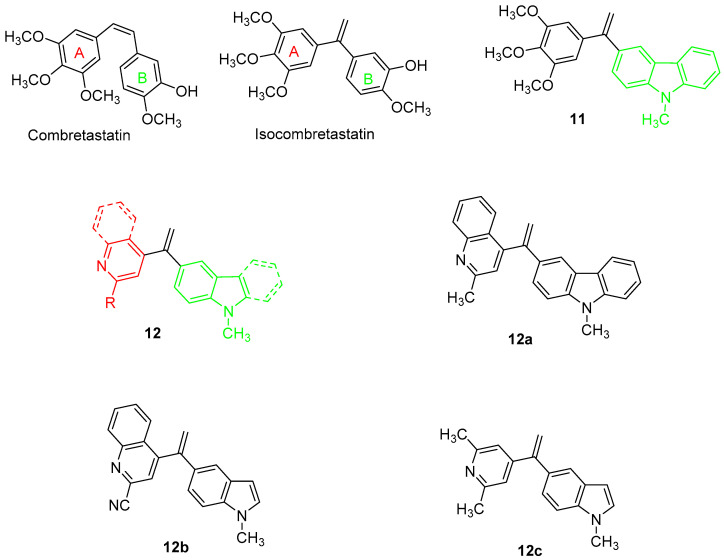
Indole-based tubulin polymerization inhibitors (**11**–**12**) in GBM.

**Figure 4:**
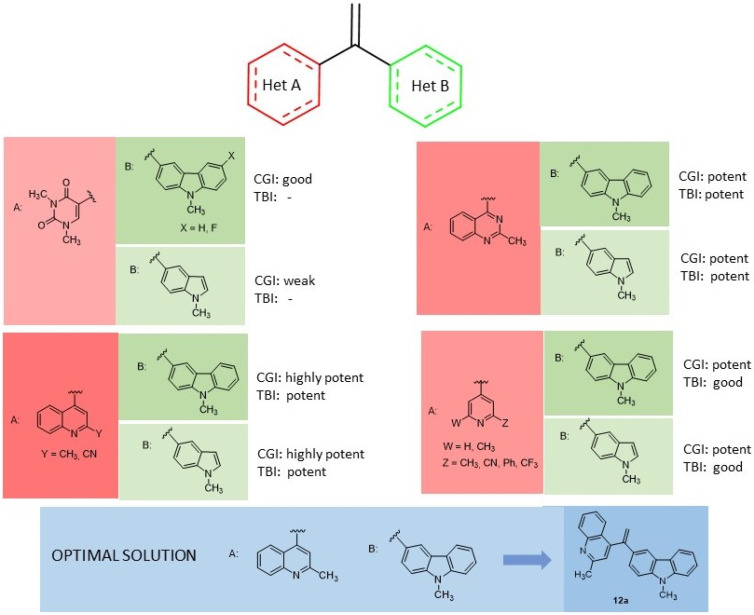
SAR [66] summary for HCT 116 cell growth inhibition (CGI) and tubulin polymerization inhibition (TBI) of indole based derivatives **12**.

In 2019, a series of bis-heterocyclic analogues of isocombretastatin were reported [66]. In previous SAR studies, the 3′,4′,5′-trimethoxyphenyl pattern, present in combrestatin and isocombretastatin as A-ring, resulted in an important requirement for optimal tubulin polymerization inhibition, whereas structural modifications of the B-ring were well tolerated [67]. Replacement of the isocombrestatin B-ring with heterocycles such as pyridine [68] and carbazole [69] (**11**, Chart 4) resulted in affording comparable activities rather than a methoxyphenol nucleus. Moreover, it was evidenced that the use of indole on the B-ring gave good results [70]. The more challenging replacement of the trimethoxyphenyl group of isocombrestatin with heterocycles such as quinoline [71] and quinazoline [72] were invaluable. In this context, 17 isocombreastatin analogues with general formula **12** (Chart 4, Figure 4), were investigated [66]. In these compounds, the trimethoxy A-ring was replaced by a pyrimidinedionyl, a quinolinyl, a quinazolinyl, or a pyridinyl moiety, while the B-ring was replaced with a carbazolyl or an indolyl moiety. Among all the new derivatives, compounds **12a–c** (Chart 4) showed very high antiproliferative activity against five different cancer cell lines including the GBM cell line, U87MG, in the range 0.81–61.9 nM. These results confirmed that the replacement of both the aromatic A- and B-rings of isocombrestatin by quinaldine and 2,6-dimethylpyridine (ring A) and by carbazole and indole (ring B) was successful. In particular, the most interesting compound, **12a**, was found to efficiently inhibit tubulin polymerization, with an IC_50_ of 2.0 μM (value comparable to that of combrestatin and isocombrestatin), by targeting the colchicine-binding site of tubulin. Moreover, **12a** was able to arrest the cell cycle in the G2/M phase at a 10 nM concentration and, interestingly, showed high antiproliferative activity in P-glycoprotein (PGP) overexpressing multidrug-resistant cells lines (K562-R and HT-29). Thus, **12a** results are of high clinical significance because PGP expression is mostly associated with chemotherapy clinical resistance. Finally, thanks to its favorable physicochemical properties, compound **12a** was permeable to the BBB, and it also showed good antiproliferative activity against the human GBM-cancer cell line U87MG. All these properties make compound **12a** a promising potential candidate for the treatment of GBM [66].

### 2.3. Indole-Based Inhibitors of the p53 Pathway 

The insurgence/progression of GBM, as well as the related chemoresistance, have often been attributed to the altered functioning of the p53 tumor suppressor derived by modifications within the p53 signaling pathway, including the overexpression of Murine Double Minute-2 (MDM2) [73,74]. MDM2 is an oncoprotein and it is the main physiological negative regulator of p53. It can bind to the p53 transactivation domain and interfere with p53 transcriptional regulatory mechanisms. Moreover, MDM2 is an E3 ubiquitin ligase promoting p53 proteasomal degradation. For all these reasons, inhibition of p53-MDM2 interaction and the consequent reactivation of endogenous p53 activity could represent a useful therapeutic strategy for the treatment of GBM [73,74]. 

MDM2 accumulates at high concentrations in cancer cells, including GBM cells, so that p53 availability is often reduced due to p53-MDM2 binding. MDM2 inhibitors are an attractive anticancer strategy for blocking p53-MDM2 protein–protein interaction, thus inhibiting the cell growth in various cancers. Anyhow, the efficacy of these inhibitors in GBM still deserves a further in depth investigation [75,76]. 

**Chart 5:**
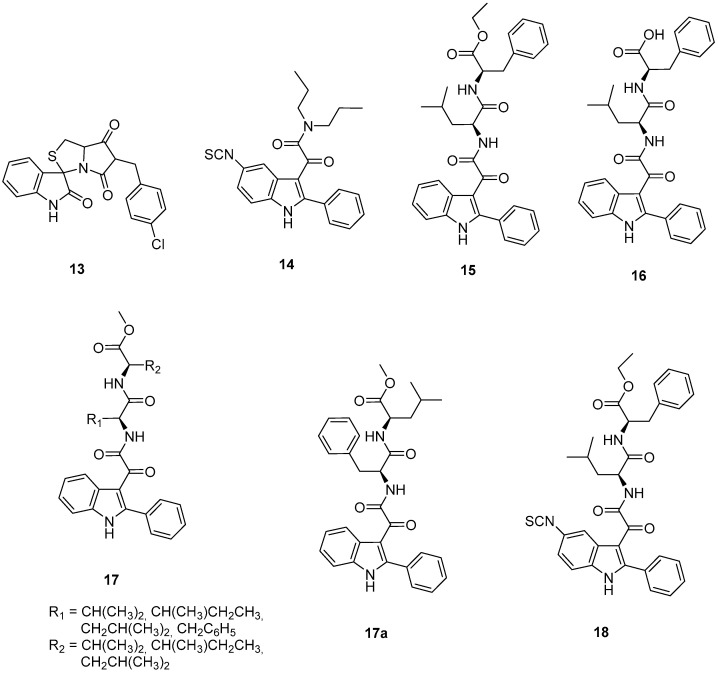
Indole-based murine double minute-2 (MDM2) inhibitors (**13**–**18**).

In this context, in 2013 a novel spirooxoindolepyrrolidine MDM2 inhibitor, **13** (Chart 5) [77], was investigated to assess its activity on human GBM cell lines [75]. Compound **13** was able to block p53–MDM2 interaction inducing a time-dependent increment of p53 expression due to the release of p53. Compound **13** was also reported to induce cell cycle arrest and apoptosis, thus effectively inhibiting human GBM cell growth in vitro. In vivo, the administration of **13** in the human xenograft GBM mouse model brought p53 activation, arrest of cell proliferation and induction of apoptosis in tumor tissue. Interestingly, **13** was non-toxic both in an in vitro healthy human cell model and also in an in vivo mouse model. Moreover, the co-administration of **13** with temozolomide produced a synergistic inhibitory effect on GBM cell viability in vitro, thus indicating a possibility of reducing the dose of temozolomide in the treatment of GBM [75]. 

The mitochondrial translocator protein (TSPO) has been found to play a key role in several cellular processes, such as cell life/death, and TSPO ligands have been demonstrated to be valid candidates for the treatment of several diseases, including cancer [78,79]. It is well known that there is no correlation between TSPO ligands’ binding affinity and their in vitro efficacy. This not only makes it difficult to assess structure–activity relationships for the discovery of new ligands, but also questions the specificity of the observed effects. In this context, the concept of residence time (RT), i.e., the time that a ligand spends on its target, has to be considered, as it is a parameter capable of influencing the pharmacological activity of the ligand itself. Taking this concept into account, the effects on the life/death processes of GBM cells produced by a reversibly binding TSPO ligand and a covalently binding one (compound **14**, Chart 5), both belonging to the class of phenylindolylglyoxylamides (PIGA), were studied in parallel [80]. The results showed that nanomolar concentrations of the irreversible compound **14** were requested to observe the same effects elicited by micromolar concentrations of the reversible PIGA ligand, highlighting that a stable ligand–receptor interaction is crucial to increase the ligands’ efficacy.

In multifactorial diseases such as cancer, therapies targeting multiple cell pathways have emerged. In this context, the same authors have exploited the idea to design dual target compounds, able to bind to TSPO and inhibit the p53–MDM2 interaction. To this purpose the PIGA class was investigated [81], taking into consideration the available SARs for binding to TSPO [82,83], and also the crystallographic p53–MDM2 interaction data [84]. This study led to the rational design of the 2-phenylindolglyoxylyldipeptide **15** and its acid analogue **16** (Chart 5), both able to efficaciously dissociate the p53–MDM2 complex, with IC_50_ values in the nanomolar range (IC_50_ values of 11.65 ± 0.49 nM and 202.0 ± 21.2 nM for **15** and **16**, respectively). 

In particular, compound **15** was shown to be about ten times more potent than the reference MDM2 inhibitors, nutlin-3 [85] or **13** [75], dissociating the MDM2–p53 complex with IC_50_ values of 108.0 ± 4.5 nM and 121.7 ± 14.5 nM, respectively. GBM cell incubation with compounds **15** and **16** resulted in a dose-dependent increase in p53 protein levels, ascribed to a decreased interaction between p53 and MDM2, and a reactivation of p53 functions. Moreover, in GBM cells, these compounds caused mitochondrial membrane potential (Δψm) dissipation, emphasizing that the dual targeting of MDM2 and TSPO amplifies the mitochondrial permeability transition pore (MPTP) opening, and a dose dependent cell viability inhibition. These effects are exerted by compounds **15** and **16** with higher potency with respect to the single target reference standards (PK11195 for TSPO and nutlin-3 for p53-MDM2) singularly applied, confirming the synergistic effect due to the concomitant modulation of the two targets [75,81,86].

**Figure 5:**
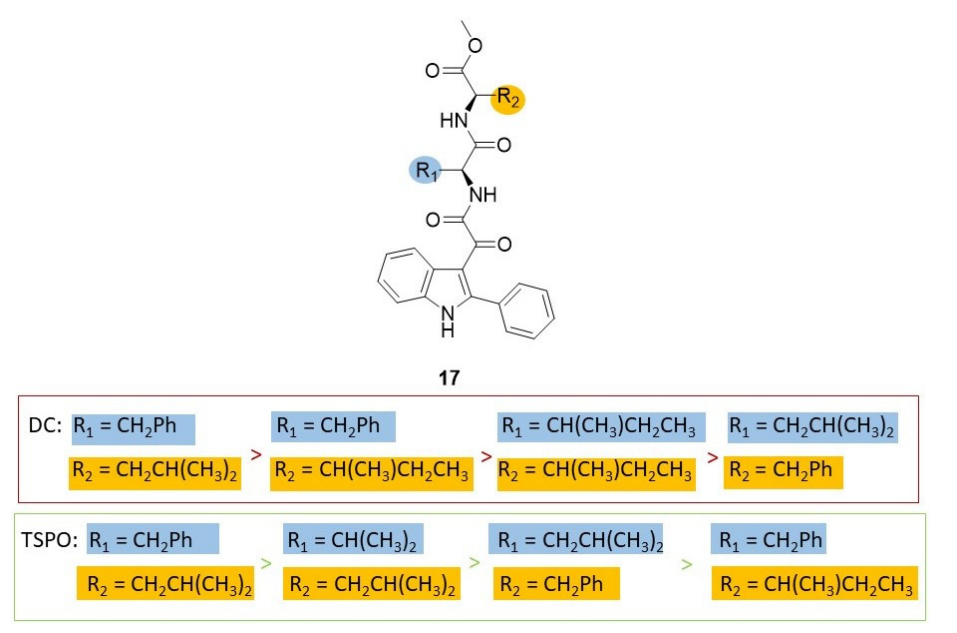
SAR [87] summary for dissociation of human p53/MDM2 complex (DC) and binding affinity to TSPO (TSPO) of indole based derivatives **17**.

Subsequently, a lead optimization process was carried out with the development of some further PIGA derivatives (general formula **17**, Chart 5, Figure 5) with different dipeptides linked to the glyoxylyl moiety [87]. As observed in the three-dimensional complex of MDM2–**15** in the MDM2 N-terminus region, in the Leu26 as well as Phe55 pockets, either aromatic or aliphatic residues were present; thus, in series **17** the terminal benzyl group of the lead compound **15** was converted into aliphatic side chains of different length and the Leu side chain into other aliphatic chains or into a benzyl group. Among the compounds of the new series, **17a** (Chart 5) emerged as the most potent dual inhibitor of p53–MDM2 interaction (IC_50_ 4.3 ± 0.6 nM)—TSPO ligand (K_i_ 87.2 ± 6.8 nM). Compound **17a** was able to reactivate p53 function and inhibit cell growth of GBM cells through cell cycle arrest and apoptosis. It was ineffective on a GBM cell line expressing mutant p53, whereas it was able to affect the viability of GSCs resistant to therapies and responsible for GBM recurrence. Finally, **17a** was preferentially active toward tumor cells compared to healthy ones [87]. 

As highlighted above, reversible drugs may not be able to maintain their therapeutic effect over time by favoring the activation of alternative signaling pathways that escape their action causing resistance, a dual-targeted compound **18** (Chart 5) has been developed, based on the structure of derivative **15**, able to produce a long-lasting binding profile to TSPO and MDM2 [88,89]. Specifically, compound **18** features a 5-isothiocyanate group able to covalently bind SH or NH groups of the target proteins, thus binding to TSPO and MDM2 in a covalent manner, with K_i_ values of 108 ± 10 nM, and IC_50_ of 6.81 ± 0.79 nM, respectively. Moreover, compound **18** inhibited GBM cell growth to a greater extent and with long-lasting effects with respect to the reversible analogue **15**, evidencing that the irreversible TSPO/MDM2 dual-targeting may represent an interesting alternative to overtake the time-limited effects of traditional drugs for GBM [89]. 

Some metals different from platinum, exploited in complexes with drugs in clinical use worldwide [90,91,92], were attractive as their various oxidation states, stability and coordination geometries, as well as thermodynamic and kinetic characteristics, may contribute to selectively bind biological targets, triggering specific cellular responses and, consequently, pharmacological effects [93,94,95]. In particular, ruthenium-based complexes with a direct metal–metal bond and a mixed valence (II,III) [96,97] have been exploited in cancer therapy because, by anchoring ligands with antiproliferative activity, it is possible to obtain the multiple release of the active molecules in the tumor site, but also the exploitation of the combined pharmacological effects of active ligands and ruthenium [98,99,100,101].

**Chart 6:**
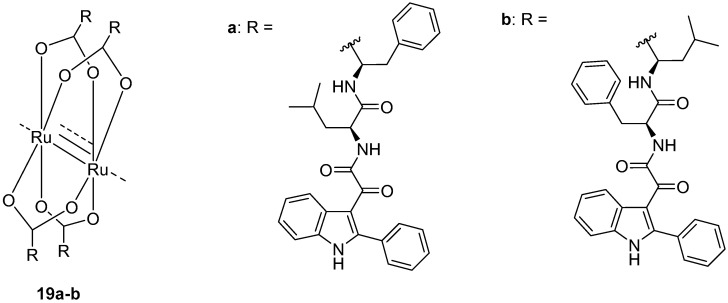
Indole based murine double minute-2 (MDM2) inhibitors (**19**) in GBM.

In this context, two Ru_2_ (II,III) complexes, **19a–b** (Chart 6) obtained from compounds **16** and **17a**, respectively, were synthesized and thoroughly characterized [102,103]. Compound **19a** was completely inactive in GBM models due to its high chemical stability: the steric protection of the dipeptidyl chain prevents the release of the active ligand by the metal and, consequently, the expected antitumor effects [102]. Instead, complex **19b** [103], characterized by the presence of four molecules of compound **17a** as ligands coordinating the Ru_2_ core, when assayed on U87MG cells, showed a cytotoxic potency lying in the µM range (IC_50_ 25.5 ± 5.7 µM). Complex **19b** thus resulted in being more reactive, and thus more active as an anticancer agent compared to its isomer **19a**, reasonably thanks to the increased accessibility of the Ru_2_ core to the attacking nucleophiles. In fact, the substantial difference between the two isomers **19a** and **19b** resides in the position of the phenyl substituent which in **19a** is in the α-position with respect to the carboxylic group, resulting in a greater steric obstacle on Ru_2_ with consequent shielding from nucleophilic attacks by the target proteins.

## 3. Conclusions

Glioblastoma (GBM) is still one of the most recurrent and aggressive CNS tumors with a poor prognosis due its unique molecular characteristics; these are the presence of a population of stem-like cells (glioma stem cells, GSCs) with ability of self-renewal and tumorigenicity that makes GBM resistant to chemotherapy and radiotherapy. Moreover, the propensity of GBM cells to infiltrate/invade the adjacent normal brain tissues and along blood vessels makes it difficult to fully resect the tumor and limits the effect of local radiotherapy. A poor prognosis for GBM is also due to the existence of the BBB making it difficult for drugs to enter the CNS and achieve sufficient effective concentration, and to a still poor knowledge of tumor-intrinsic dominant signaling pathways as well as tumor-specific antigenic profiles.

In the last decades, improved genomic, epigenetic, transcriptomic, and proteomic characterization of GBM and of the brain microenvironment and immune system interaction have helped in developing innovative clinical trials. In this context, the efficacy of biomacromolecules is increasingly recognized in the search for new therapeutic strategies for GBM such as immunotherapy, including immune checkpoint blockade, chimeric antigen receptor T (CART) cell therapy, oncolytic virotherapy and vaccine therapy.

To date, many researchers are trying to use synergistic immunotherapies as a good therapeutic prospect for GBM treatment. Oncolytic viruses were also shown to promote tumor regression by inducing immunogenic cell death mainly in tumor cells: some of them are approved for the treatment of certain malignant gliomas, even if further studies have to be carried out to assess which GBM patients would benefit the most. The personalized vaccination trials were quite feasible and with a certain therapeutic potential, with autologous dendritic cell vaccines resulting in superior therapeutic efficacy compared to peptide vaccines; surely, this approach to GBM treatment warrants further in-depth studies.

In the treatment of GBM, small molecules, due to their rather simple structure, have a greater advantage in entering the CNS. In addition, the cost of the small molecules is much lower than that of biological macromolecules, making them more attractive to patients. Moreover, the discovery of more targeted small molecules with excellent BBB permeability necessary for innovative treatment of GBM may aid in silico methods such as virtual screening (VS), that can rapidly provide a set of biologically active compounds in an economically efficient manner. Very recently, the employment of artificial intelligence methods such as deep and machine learning has added value in small-molecule drug discovery. These methods give access to better success rates by overcoming the intrinsic hurdles connected with standard ligand- and receptor-based methods. Thus, although there is still much to understand on the mechanisms and biomarkers of GBM, and intense research and clinical development are required to optimize the available molecules and to overcome their potential side effects, with the help of these innovative in silico discovery technologies small molecules may represent the main direction for the development of drugs for an efficient and effective treatment of GBM.

The indole nucleus is considered a privileged structure in medicinal chemistry and, overall, indole compounds have emerged as a valuable tool in the ongoing battle against life-threatening diseases, such as cancer. In fact, the vast amount of indole compounds entering the pre-clinical and clinical study phases highlights the significant progress achieved in this area. 

In this context, the present review summarized the state of the art on indole-based compounds in the pre-clinical and clinical stages of development against GBM, mainly focusing on the most recent advances in medicinal chemistry of indole derivatives with anti-GBM activity (in vitro and/or in vivo), reported between 2013 and 2022. 

In the treatment of GBM, most of the molecular targets studied in recent years involve kinases, and in particular PI3K, PDK1, CK2, c-Src, Akt/PKB, FAK, VEFRG, and EGFR. Other enzymes, signaling pathways and cellular processes are also involved, including G-quadruplex, HDAC, HSP90, microtubule, p53, among others. The indole-based derivatives as anti-GBM agents, herein reported, act mainly on three targets which are essentially kinases, tubulin and p53. In most of the studies performed, in vitro studies in GBM cell lines, mainly U87MG and U118MG, were involved, and some of the indole-based compounds reported were found to have promising activity.

With this review we aimed to provide an outlook on existing data of the indole-based derivatives that might help the medicinal chemists in the future design and development of more efficient and selective novel indole-based leads for in vitro and in vivo studies, so that new more effective GBM drug candidates can be unveiled and introduced to the market and into clinical usage.

## Data Availability

Not applicable.
